# Linear Low-Density Polyethylene Enriched with Graphene: Effects on Its Rotational Molding and Properties

**DOI:** 10.1021/acsomega.5c06155

**Published:** 2025-09-25

**Authors:** Francisco Pereira de Araújo Júnior, Enzo Erbisti Garcia, Gerson Alberto Valencia Albitres, Luis Claudio Mendes, Peter Martin, Mark McCourt, Bronagh Millar, Paula Douglas

**Affiliations:** † Instituto de Macromoléculas Professora Eloisa Mano, Centro de Tecnologia, 28125 Universidade Federal do Rio de Janeiro, Avenida Horácio Macedo, 2030, Bloco J, Ilha do Fundão, Rio de Janeiro 21945-970, Brazil; ‡ Polymer Processing Research Centre, Queen’s University Belfast, University Road, Belfast BT7 1NN, Northern Ireland, U.K.

## Abstract

Graphene (GP) belongs to a class of structured carbon compounds with outstanding properties that address its application in energy storage devices, water filtration, biomedical fields, and so on. To take advantage of its high thermal conductivity, this work was thought to study the effect of GP on the rotational molding and properties of linear low-density polyethylene (LLDPE). First, the better molding conditions were determined by different internal peak air temperatures. Once chosen, LLDPE was enriched with a masterbatch containing GP at different contents. Significant energy savings in the rotational process were achieved by addition of GP to LLDPE. Through the drop dart impact test, the presence of GP improved the impact strength. This finding was endorsed by the calorimetric measurement, which revealed a decrease in the LLDPE degree of crystallinity. The melt flow rate showed a slight variation with the GP content. The presence of the GP did not change the rheologic behavior of the composites. Even in very low content, WAXD and Raman spectroscopy revealed the presence of GP in the composites. Thermal conductivity of the composites increased when GP was added to LLDPE which could explain the energy savings in the composites’ processing and their impact property. As a consequence, the increment of thermal conduction affected the cooling rate of LLDPE chains, leading to some extent to the amorphization of the polymer matrix.

## Graphical abstract

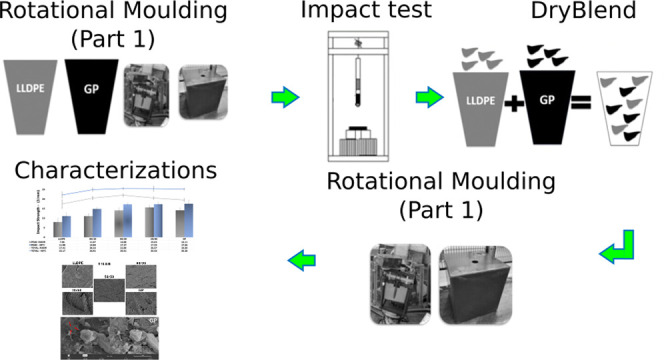

## Introduction

Society needs to promote technological advances to improve the quality of life of its population. Therefore, research and development of new materials are pillars of support in various applications. The civil construction industry, for example, has been using more and more new materials, seeking improvement of characteristics such as increased mechanical resistance and lightness. To achieve these objectives, the integration of properties pertinent to different materials appears as a reality that opens space for the development of new composites with outstanding properties.

Polymeric composites are examples of materials that enable associations of properties and can be used to solve various problems. However, there are still limitations that prevent an increase in production scale, generally associated with the degree of difficulty of processing and/or associated costs.^1,2^

Rotational molding is a plastic transformation process that can benefit from the use of structured carbon compounds. This type of manufacturing is considered financially advantageous for obtaining large and complex parts with a uniform thickness. However, the production time is usually long, consisting of four steps: loading (stage of placing the material in the mold), heating, cooling, and demolding (removal of the part).^3–5^ Usually, polyolefins are used in rotational molding. Aiming to use the concept of circular economy, Cestari et al. molded virgin and recycled polyethylene blends at different proportions by compression and rotational molding. It was suggested that the wall thickness reduction of the parts and size of the ground achieve better impact performance.^6^

A greater number of studies are addressed to the effect of adding micro- and nanofillers on the polyolefins matrix and rotational molding. Rotomolded specimens based on composites of polyethylene and coconut fibers were studied. The authors pointed out that the impact strength decreased with increasing of fibers content.^7^ Halloysite nanotubes were embedded in the linear low-density polyethylene (LLDPE) matrix. According to the authors, the improvement of mechanical and thermal properties addresses the composite for rotational molding applications.^8^ Microtalc and nanoclay as nucleating and reinforcing agents, respectively, were incorporated into polyethylene. Specimens were rotomolded by a laboratory-scale rotational machine. Enhancement of foam density and decrease of cell size were attributed to the synergistic effect of the fillers.^9^ Hamidi et al. studied by modeling the process of melting of the grains of thermoplastics when submitted to rotational molding. The study considered that the densification occurred during the first stage while the second stage, the migration of air, was controlled by diffusion.^10^ An investigation on how fibers alter the densification of the polymer grains and its action on the arising and the vanish of air bubbles in rotational molding of polyethylenes was conducted by Castellanos and collaborators. The results revealed that although heating parameters and particle size affect bubble morphology and its disappearance, the fibers did not significantly change it. Also, polymer–fiber characterization plays a crucial role in sintering and densification of the grains.^11^ Rotomolded composites of polyethylenes embedded with halloysite were investigated by Höfler and collaborators. The effect of the filler on the flow and mechanical properties were evaluated. Even for small amount of halloysite, all polyethylenes showed decrease in the impact behavior.^12^ Using rotational molding, Zepeda-Rodríguez et al. prepared composites of polyethylene filled with carbon nanofibers at contents of 0, 0.01, 0.1, and 1.0 wt %. Monitoring the internal air temperature, the authors registered the melting and crystallization of the polymer matrix which were endorsed by calorimetric measurements.^13^ Mineral dust—ignimbrite from quarries, rich in silicon dioxide, SiO_2_—was added to the polyethylene matrix, and its outcome on energy consumption in rotational molding was assessed. It was pointed out that the reduction in oven time was around 27% for composite incorporated with 10% of ignimbrite.^14^ The efficiency of dry blending in the incorporation of expanded vermiculite in polyethylene and further rotational molding was studied by Aniśko and collaborators. The study revealed that two-step process showed significant improvements in the mechanical properties.^15^ Daryadel et al. prepared composites of LLDPE with Cloisite 30B (0, 1, and 2 wt %) for aftermost rotational processing. It was pointed out that the processing temperature was the main parameter associated with mechanical properties while the second one was the rotational speed.^16^ Yadav et al. studied the effect on the LLDPE rotational processability when it was filled with bamboo fiber. By the melt flow rate (MFR), the best compositions were those with 5 wt % (3.8 g/10 min) and 10 wt % (3.09 g/10 min) of bamboo fiber, respectively.^17^ Azodicarbonamide (blow agent) was added to recycled high density polyethylene to produce a foamed rotomolded item. The optimum result of impact strength was attained for composite with 0.1 wt % of blow agent.^18^ Graphene (GP) has been considered to be a revolutionary material. It has been gaining more and more attention, increasing its range of applications. Its special characteristics is due to its structural two-dimensional arrangement, one-atom thick sheet composed by a monolayer of sp^2^ hybridized carbon atoms which can adsorb aromatic organic compounds via van der Waals interaction and π–π electron coupling.^19,20^ Usually, the addition of GP to polymers improves mechanical and thermal properties, electrical conductivity, and dimensional stability. Lv et al. presented a review which highlighted the thermal conductivity of GP and its chemical modification to improve distribution and dispersion on the epoxy resin matrix.^21^ To overcome the challenge of improving the GP thermal conductivity in films with thick upper than 100 μm, Liu et al. applied a combination of preparation and treatment methods to attain ultrathick and highly conductive GP films.^22^ This work intended to study the action of GP when incorporated into the LLDPE matrix and its repercussions on polymer processability, thermal, and impact strength when rotational molding was applied.

## Experimental Section

### Materials

LLDPE grade 4439 and polyethylene nanocomposite containing 0.02% graphene (coded as GP) were supplied by Northeast Compound, Brazil. The two materials (particle size of 500 μm) were considered for rotational molding. Table 1 reveals some of their physical properties.

**Table 1:** Precursors’ Information

code	bulk density (g/cm^3^)	dry flow time (s)
LLDPE	0.941	24
GP	0.943	23

### Evaluation Stages

Figure 1 illustrates the flowchart of the processing methods employed for sample preparation as well as the characterization techniques utilized in the study.

**Figure 1:**
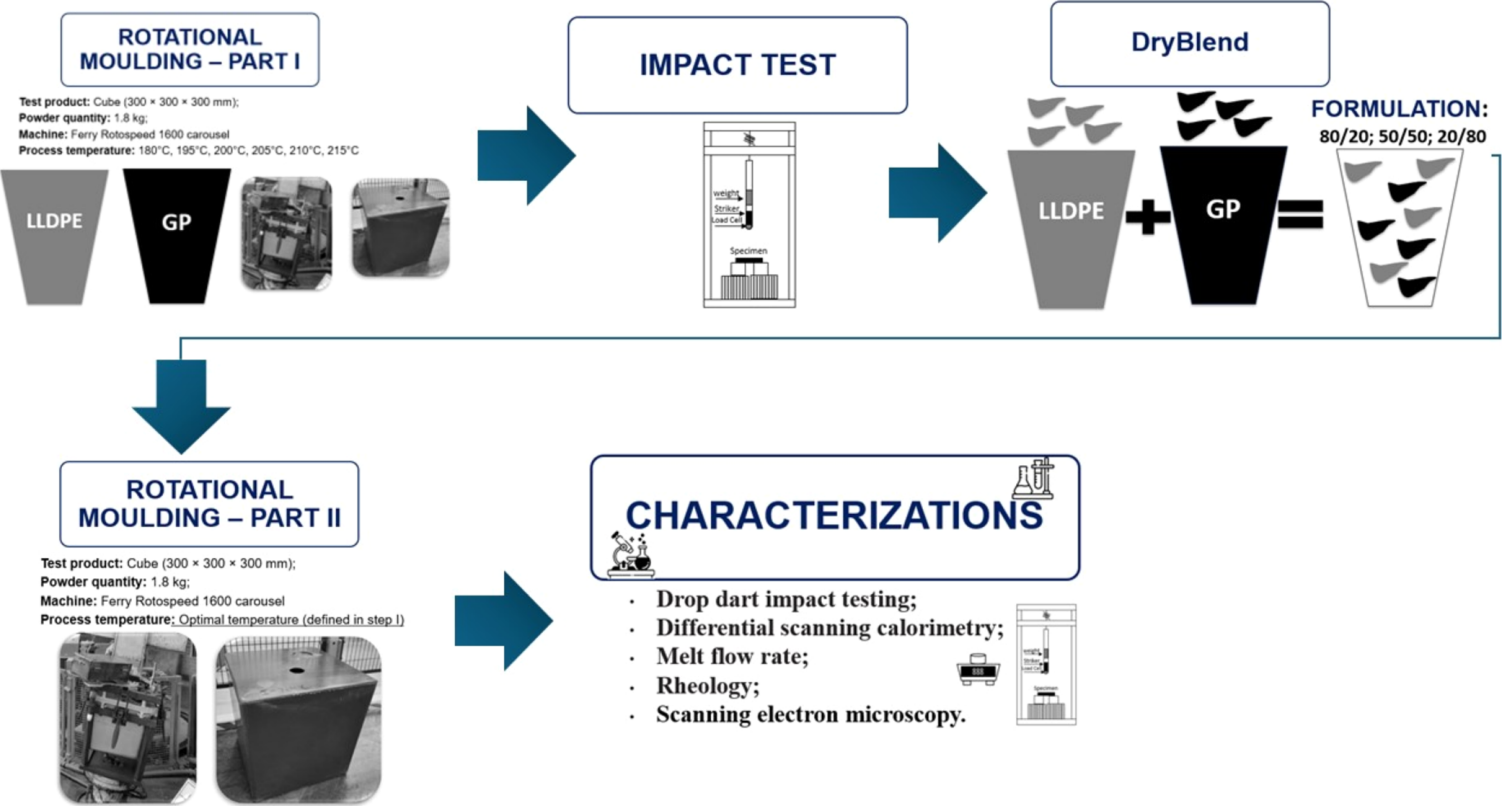
Flowchart of samples’ processing and characterization.

#### Precursors’ Rotational Molding—Part I

To achieve the ideal processing conditions of the precursors, about 1.8 kg of each one was put into an aluminum rotational molding with dimensions of 300 mm × 300 mm × 300 mm and specimens with 3 mm of thickness were prepared. The mold external temperature was set at 300 °C. The Rotopaq real-time monitoring system was used to record the variation of air temperature as a function of time inside the mold applying internal peak air temperature (PIAT) of 180, 195, 200, 205, 210, and 215 °C. The rotational molding machine type Ferry RotoSpeed 1600 carousel was used. The best process condition was chosen by considering the drop dart impact test evaluation. Once chosen, the same conditions were applied for the composite LLDPE/GP.

#### Composite Preparation: Dry-Blend and Rotational Molding—Part II

To prepare the composites, the precursors were mixed (conventional dry-mixing was applied) at different compositions, as shown in Table 2. The rotational molding condition was like that selected for the precursors.

**Table 2:** Composite Formulation

code	LLDPE (%)	GP (%)	graphene (%)
LLDPE	100	0	0.000
80/20	80	19.996	0.004
50/50	50	49.990	0.010
20/80	20	79.984	0.016
GP	0	100	0.020

### Drop Dart Impact Testing

The test was carried out according to BS EN ISO 6603-2 using a CEAST Fractovis machine at room temperature and −40 °C.

### Thermal Conductivity

To evaluate the effect of GP on LLDPE, thermal conductivity was determined indirectly at 210 °C (processing temperature), considering its correlation with thermal diffusivity. The following relationship was used:

$$\alpha =\frac{k}{\rho c_{p}}$$

where α = thermal diffusivity (m^2^/s), ρ = density (kg/m^3^), *c*_*p*_ = specific heat capacity (J/kg K), and *k* = thermal conductivity (W/m K) published by Carson and Alsowailem.^23^ The thermal diffusivity of molten polyethylene was 6.92 × 10^–7^ m^2^/s as published by Yánez et al.^24^ Density at 210 °C was 742.5 kg/m^3^ being estimated as published by Orwoll in Physical Properties of Polymers Handbook.^25^ Specific heat capacity was determined using a TA calorimeter model Q1000 following ASTM E1269.^26^ The result was expressed by the average of two measurements.

### Differential Scanning Calorimetry

Differential scanning calorimetry (DSC) was performed according to ISO 11357-3:2018 using a PerkinElmer DSC machine. The sample was heated from 30 to 200 °C, 10 °C/min, at the nitrogen atmosphere, keeping 3 min to eliminate the thermal history. After that the sample was cooled to 30 °C. Finally, the last heating was performed to 200 °C. Crystallization temperature and second melting temperature (*T*_c_ and *T*_m_) were evaluated. The degree of crystallinity (*X*_c_) was determined by considering the second melting peak. The calculation was based on the ratio of the experimental melting enthalpy and theoretical melting enthalpy of polyethylene (293 J/g).^27^

### Wide-Angle X-ray Diffraction

Crystallographic evaluation was conducted in a Rigaku Ultima IV diffractometer, with Cu Kα radiation (λ = 1.5418 Å) and experimental conditions of 40 kV, 20 mA, step of 0.05, ranging the 2θ angle from 2° to 40°.

### Raman Spectroscopy

Raman spectroscopy was performed using a Raman Microscope equipped with a laser, at a wavelength of 532 nm and a 50× lens, in the range 4000–200 cm^–1^. The vibrational modes were evaluated.

### Melt Flow Rate

The MFR was determined according to ASTM D1238 using a melt indexer (MP600-Tinius Olsen). The test parameters were 190 °C/2.16 kg.

### Rheology

The rheology was carried out in a TA rheometer, model AR-2000 with parallel plate geometry, with 25 mm of diameter, at 140 °C, strain amplitude of 10^3^, and frequency range of 10^–1^ to 10^3^. The complex moduli (storage and loss) and viscosity were evaluated.

### Scanning Electron Microscopy

A Tescan field emission scanning electron microscope, model MIRA 4 LowVac Mode UniVacTM equipment, voltage of 10 kV was used to take the scanning electron microscopy (SEM) image of the fractured surface.

## Results and Discussion

### Rotational Processing and Impact Evaluation

According to Umbare and Arakerimath, the control of PIAT is essential to ensure proper material processing and to prevent defects in the parts.^28^ Impact strength and absorbed impact energy were chosen as the property to evaluate the optimal processing conditions.^29,30^ In Figure 2, the processing conditions of the materials were evaluated by using the Datapaq system. The results demonstrated that the material containing GP rendered better the processing, attributed to its high thermal conductivity. Table 3 presents the processing curves’ area for both precursors. The energy increase represents how much energy LLDPE needs than GP. At 180 °C, the specimens presented bubbles’ formation, indicating that the temperature should be negligible. From that temperature, the curves’ area of both precursors showed continuous tendency to increase. As can be seen, above that temperature, LLDPE required more energy than GP for processing while the temperature increased. Increase energy represents the additional absorbed energy by LLDPE when compared to the GP for processing in each temperature.

**Figure 2:**
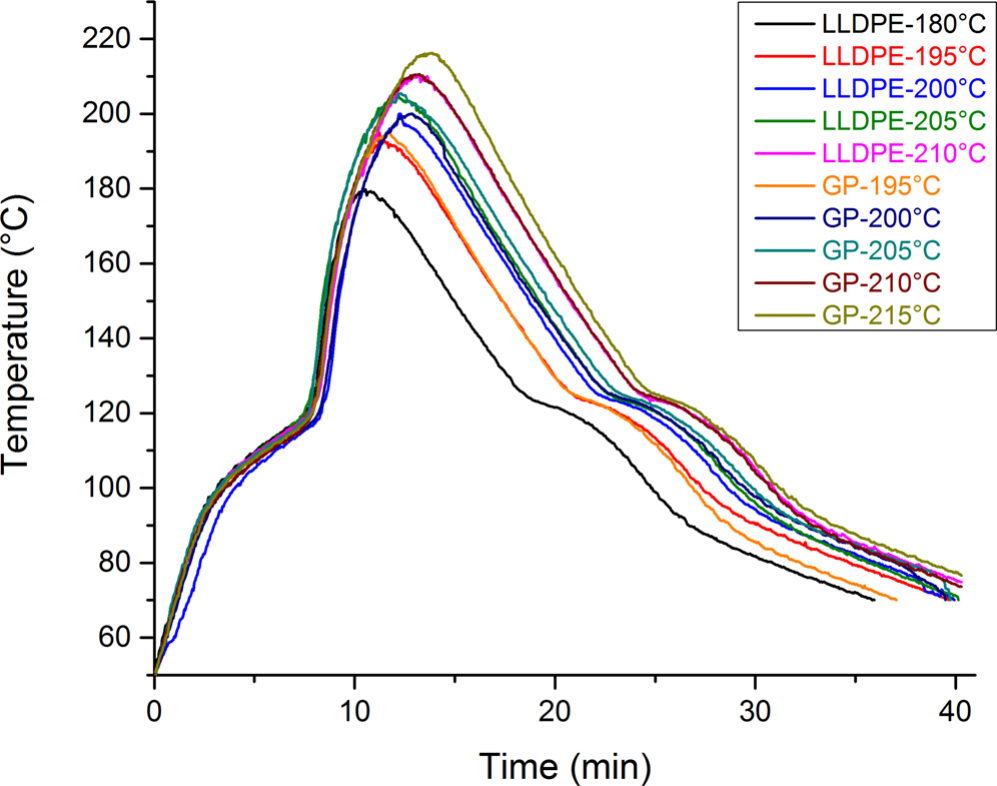
Precursors’ rotational processing curves at various PIAT.

**Table 3:** Calculation of Precursors’ Processing Curves Area

temperature (°C)	LLDPE processing curve area	temperature (°C)	graphene processing curve area
180	240.5	247.0	–2.7
195	278.5	263.7	5.3
200	285.2	262.7	7.9
205	296.8	272.1	8.3
210	318.1	290.4	8.7

To associate the best PIAT with the impact strength of each precursor, the specimens were submitted to impact assessment at −40 and 25 °C. Figures 3 and 4 show the evaluation of impact strength and absorbed impact energy. LLDPE and GP (PEAK) are related to impact strength, while LLDPE and GP (TOTAL) are associated with absorbed impact energy. At −40 °C, for both precursors, the impact strength increased continuously with processing temperature, but LLDPE tended to the stability. Progressively, for both, the absorbed impact energy increased with processing temperature. At 25 °C, for GP, the absorbed energy continuously grew while for LLPDE after 205 °C the impact strength showed decrease. For both precursors, the absorbed impact energy depicted a gradual rise. In summary, it is possible to see that impact behavior of GP is better than LLDPE. For both precursors, the best PIAT ranged between 200 and 210 °C; since below 200 °C, it was noticed worse surface finish of the specimens while above 210 °C, LLDPE started to lose impact property.

**Figure 3:**
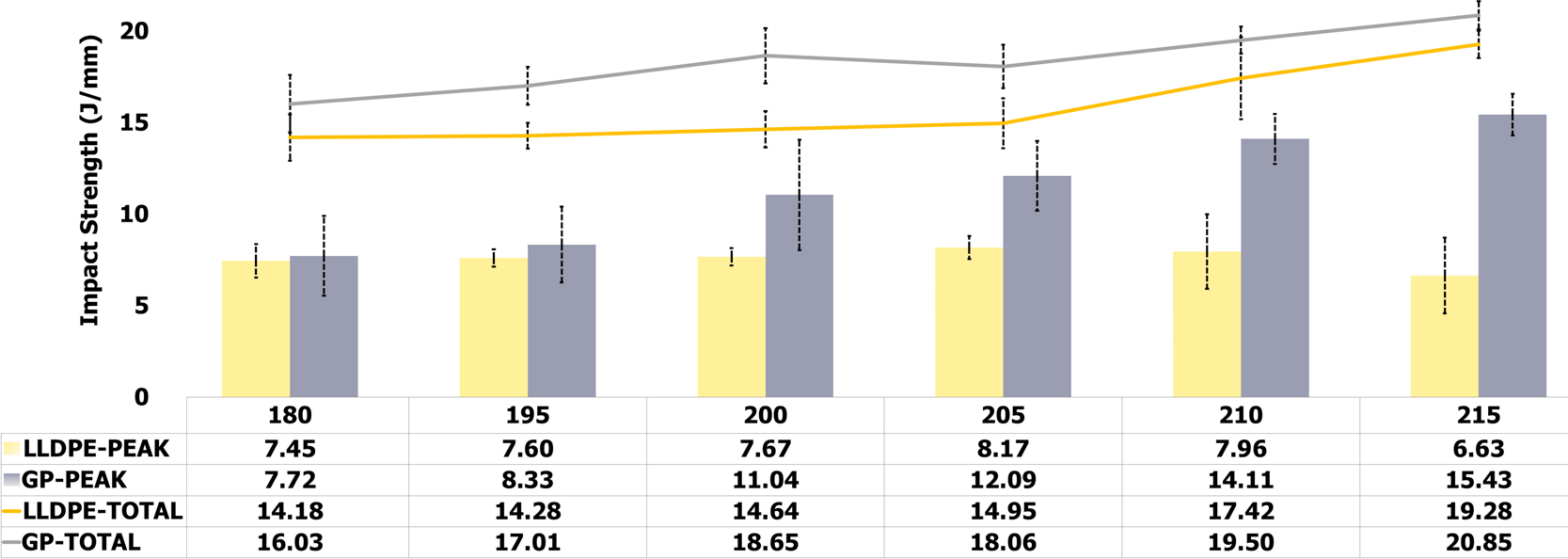
Precursors’ rotational processing curves at various PIAT.

**Figure 4:**
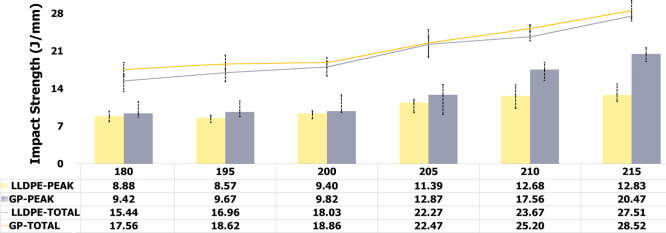
Precursors’ rotational processing curves at various PIAT.

With the view of the results, the best PIAT temperature (210 °C) was considered. Then, the composites were submitted to rotational molding. Figure 5 presents composite rotational processing curves. Clearly, the addition of GP in LLDPE promoted a decrease in the cooling process. The curves of the composites 80/20 and 20/80 are superimposed while that one for 50/50 was intermediate between those ones and precursors.

**Figure 5:**
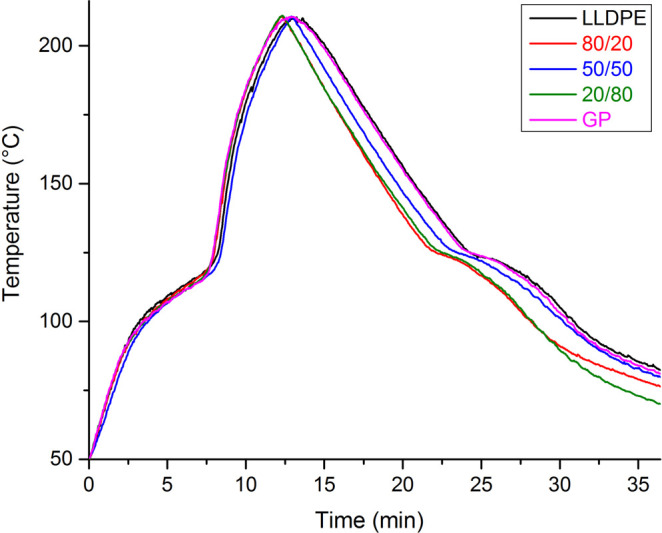
Composites’ rotational processing curves at PIAT (210 °C).

Table 4 displays the composites’ processing curves area and energy increase related to LLDPE and GP—representing the processing energy variation associated with addition of LLDPE and GP to each other. With respect to LLDPE, all composites’ processing area decreased abruptly as the content of GP enriched them. The reduction of processing energy was more economically advantageous when GP was added to LLDPE, being the best result for LLDPE/GP 20/80 (reduction around 14%). On the contrary, when GP was gathered to LLDPE, it was also useful although energy saving was less.

**Table 4:** Calculation of the Composites’ Processing Curves Area

code	processing curves’ area (KJ·s)	energy increase based on LLDPE^a^ (%)	energy increase based on GP^a^ (%)
LLDPE	318.1		
80/20	290.2	–8.8	–0.05
50/50	295.6	–7.1	–1.8
20/80	273.4	–14.1	–5.9
GP	290.4		

a Energy increase calculation: (LLDPE/GP) – (LLDPE or GP) (KJ·s)/(LLDPE) or (GP) (KJ·s) × 100.

Figure 6 exhibits the impact strength (PEAK) and absorbed impact energy (TOTAL) of the composites at −40 and 25 °C. At both temperatures, the impact strength tended to increase but at −40 °C, there was tendency to stability from 50 wt % of GP. For useful temperature (25 °C), the impact strength increased 39, 77, and 96% for the composites 80/20, 50/50, and 20/80, respectively. In general, the absorbed impact energy seems to be invariable in both temperatures. Summarizing, the low content of GP in GP precursor imputed better impact strength in the LLDPE matrix. Besides, it led to significant energy saving. These results were achieved due to the GP thermal conductivity which make the heat transfer easy through LLDPE chains during cooling of rotational molding possibly, leading to the variation of LLDPE degree of crystallinity.

**Figure 6:**
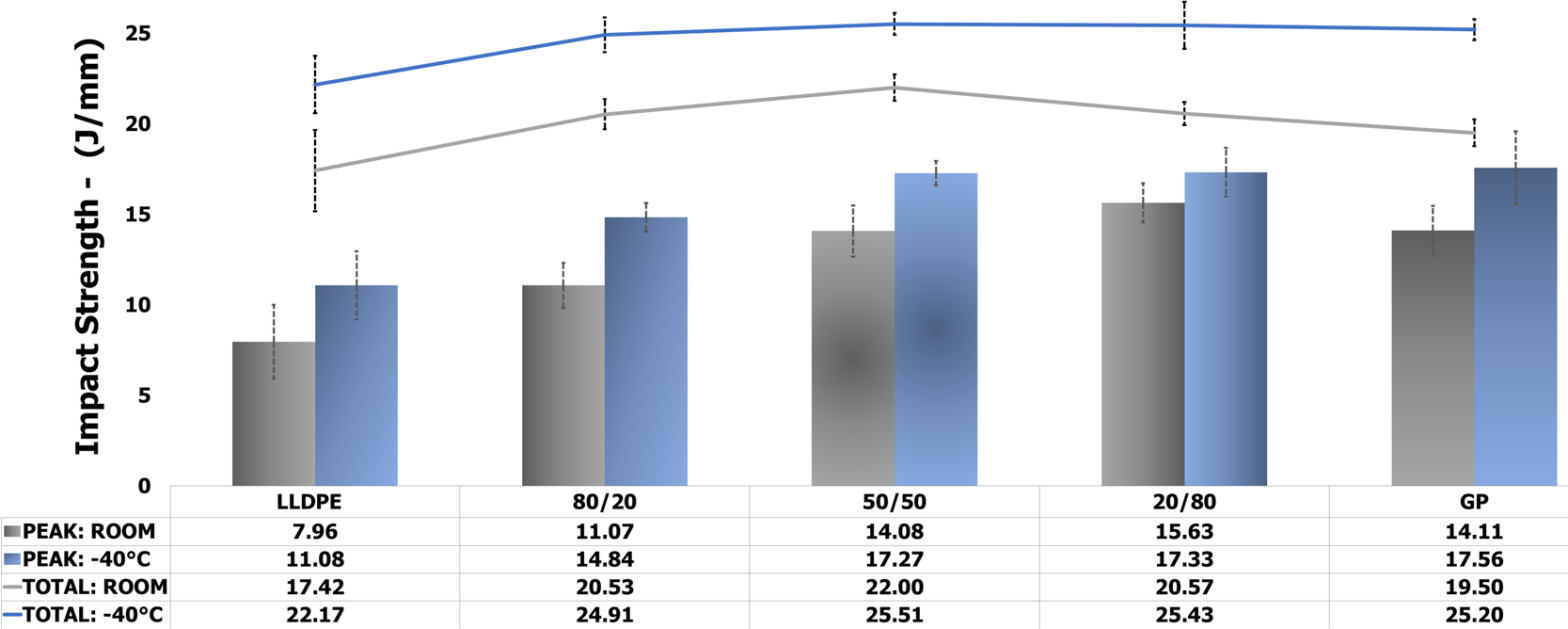
Precursors and composites’ impact strength and absorbed impact energy (−40 and 25 °C).

### Thermal Conductivity

Table 5 exhibits the values of the thermal conductivity. As detailed in the experimental section, thermal conductivity was determined indirectly by correlating it with thermal diffusivity. Although nonlinear, the addition of GP to LLDPE increased the composites’ thermal conductivity. This result is consistent with those found for sample processing.

**Table 5:** Samples’ Specific Heat Capacity (*C*_*p*_) and Thermal Conductivity (*k*)

sample	*C*_*p*_ (J/kg K)	*k* (W/m K)
LLDPE	2575	1.32
80/20	2860	1.47
50/50	2685	1.38
20/80	2620	1.35
GP	2900	1.49

### Differential Scanning Calorimetry

Figures 7–9 highlight the cooling curves, melting curves of first and second heating cycles, and their amplification, respectively. For all samples, the cooling temperature (*T*_c_) was the same. On the contrary, the melting temperature (*T*_m_) varied from the first heating cycle to the second one. Differences were better viewed when the range of melting was amplified. The first heating curves contain the thermal history of the rotational processing. The melting peak of LLDPE, GP, and LLDPE/GP (50/50) is split into two maximum. On the contrary, the composites 80/20 and 50/50 show a single peak; that one related to the composite 80/20 was more enlarged. When the thermal history was eliminated, all samples revealed a unique melting peak in the range of 127–128 °C. Table 6 exposes the values of the degree of crystallinity (*X*_c_) in the first heating cycle. Sharply, for the composites, when GP was added to LLDPE, *X*_c_ decreased in the percentage of 12, 16, and 19%, respectively. The finding was imputed to the improvement of thermal conductivity promoted by GP into the LLDPE matrix. According to Yang et al., GP-based material possesses excellent thermal conductivity, hydrophobicity, and mechanical properties and represents a new option for electronic devices.^31^ Zhou et al. developed a technique concerning in situ growth of GP onto carbon fibers for improvement of epoxy composites’ properties. An increase of 251% in thermal conductivity was achieved.^32^ Jalali et al. prepared composites as foam by 3D-printing based on a polyvinylidene fluoride/GP/foam agent composite. Better thermal conductivity was attained in the composite with 7% of GP.^33^ The thermal conductivity of GP influenced the cooling process during the rotational molding. The improvement of thermal conductivity leads to better thermal exchange inside the specimen. As consequence, the crystallization process was affected, resulting in the decrease of degree of crystallization. It is a kind of amorphization provoking improvement of the impact properties of the specimen.

**Figure 7:**
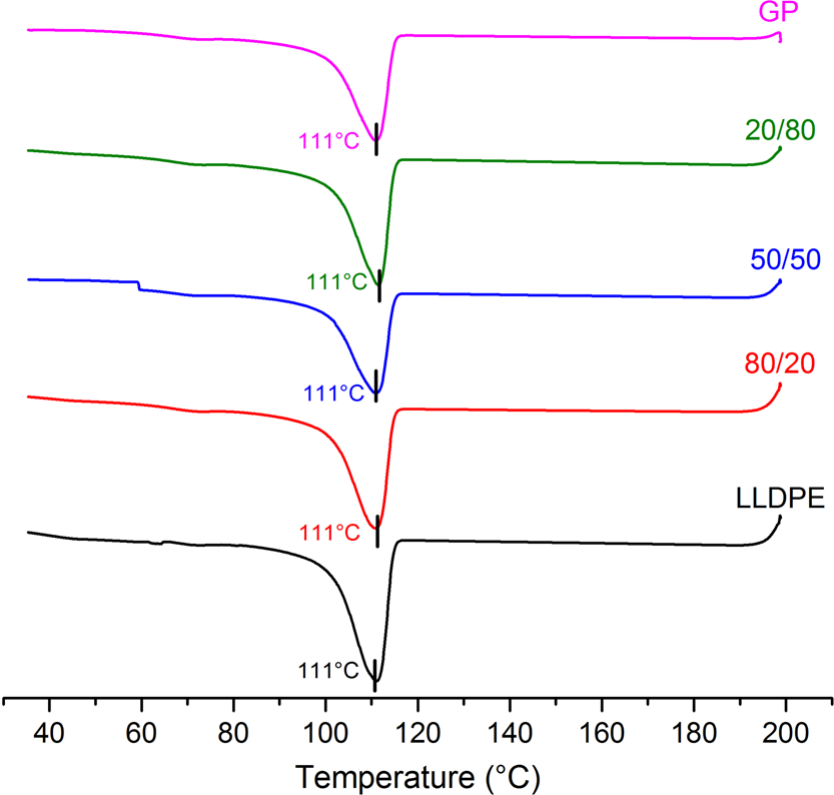
Cooling curves of the precursors and composites.

**8:**
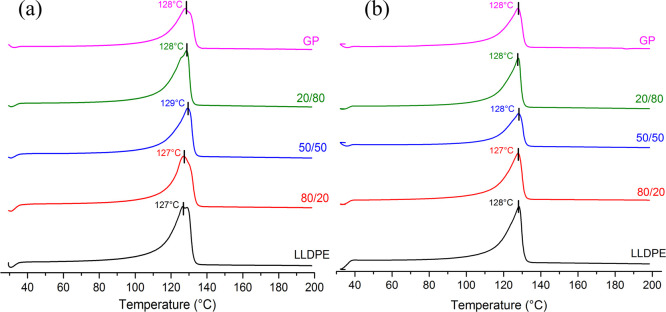
First (a) and second (b) heating curves of the precursors and composites.

**Figure 9:**
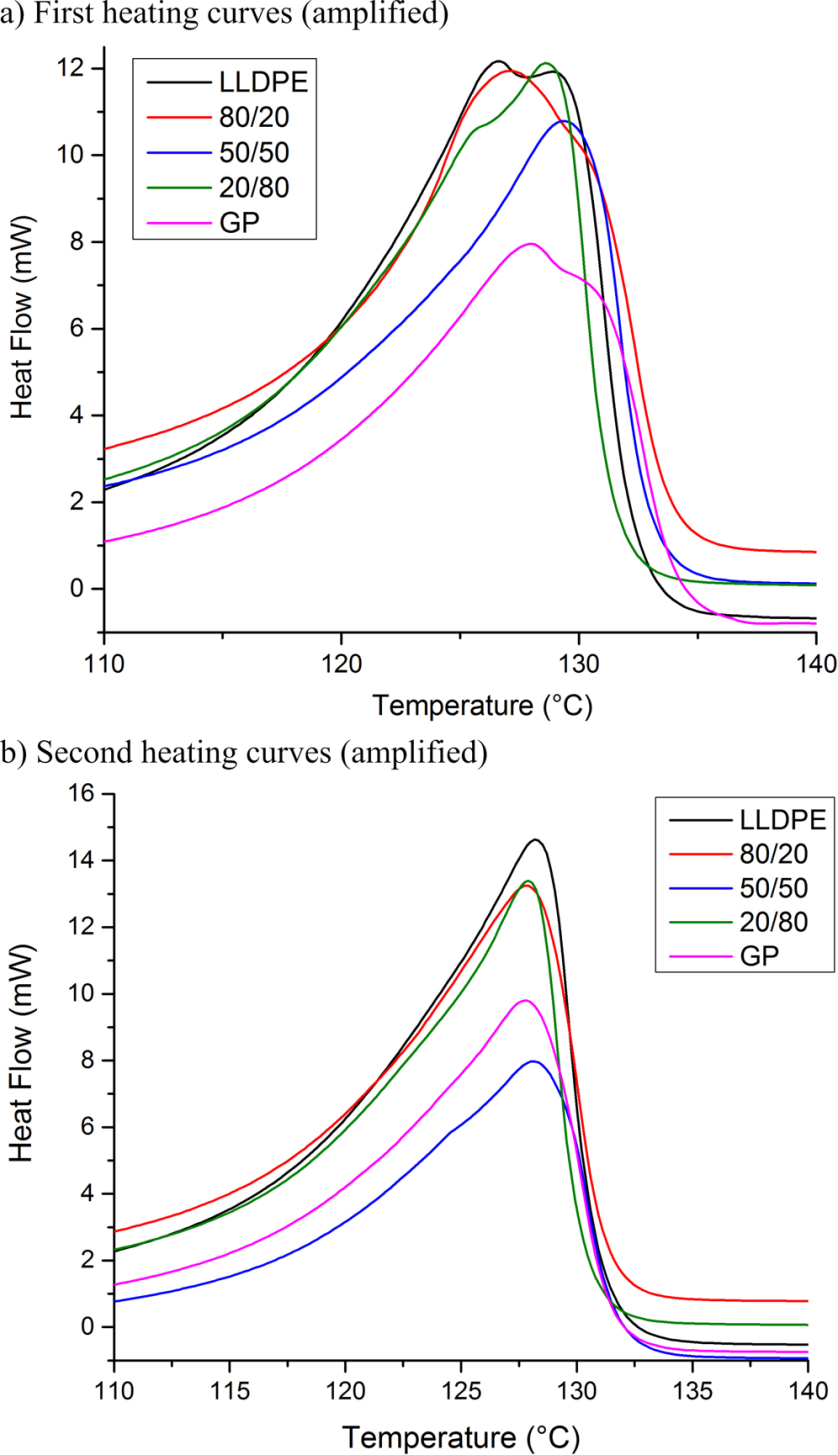
(a,b) Amplification of first and second heating curves of the precursors and composites.

**Table 6:** Precursors and Composites’ Calorimetric Data Relating to the First Heating Cycle

code	*T*_c_ (°C)	*T*_m_ (°C)	*X*_c_
LLDPE	111.1	126.7	58.1
80/20	110.9	127.2	50.8
50/50	111.0	129.4	48.8
20/80	111.4	128.5	46.7
GP	110.9	127.9	38.5

### Wide Angle X-ray Diffraction

Figure 10 shows the diffraction patterns of the samples. For all, the diffraction angles of the orthorhombic form of polyethylene were observed at 2θ around 21° and 24°, corresponding to the crystalline planes 110 and 200, respectively, but except for LLDPE, the GP’s diffraction angle was detected at 2θ around 26° (see the amplified region), related to crystalline plane 200.^34,35^ It is worth highlighting that the 20/80 sample presented new diffraction angles between 14° and 16°. There are several publications dealing with the occurrence of transcrystallization in polymer mixtures, compatibilized or not, micro- and nanocomposites, among others. Bateni et al. investigated the effects of remolding temperature and thermal annealing on the properties of nanocomposites of maleic anhydride grafted polyethylene [PE-g-MA] filled with electrospun nanofibers of polyamide-6 (PA-6) and polyamide-6/GP oxide (GO/PA-6). The authors pointed out that calorimetric results revealed a slight increase in PE crystallinity due to the induced crystallization on the surface of the nanofiber. Also, they observed that a crystalline region with thickness of 20 μm was developed on the nanofiber surface or nanofiber rich region, named transcrystalline region.^36^ Varga and Karger-Kocsis published an article devoted to clarifying the difference between transcrystallization and shear-induced cylindritic crystallization in fiber-reinforced polypropylene composites. They stated that the transcrystallization is a kind of physical coupling induced by heterogeneous nucleation.^37^ Wang et al. and Qiu et al. studied the effect of calcium carbonate and talc on the development of the transcrystallization process in PP composites.^38,39^ The emerging diffraction angles at 14° and 16° could be attributed to the development of transcrystallization; since by DSC evaluation, the 20/80 sample maintained *T*_c_ of LLDPE but registered an increase on its *X*_c_ when compared to the GP sample. Those diffraction angles could be associated with monoclinic and/or hexagonal crystal forms of LLDPE as recorded by Feng et al. in their study on stretching of LLDPE at different strain rates.^40^

**Figure 10:**
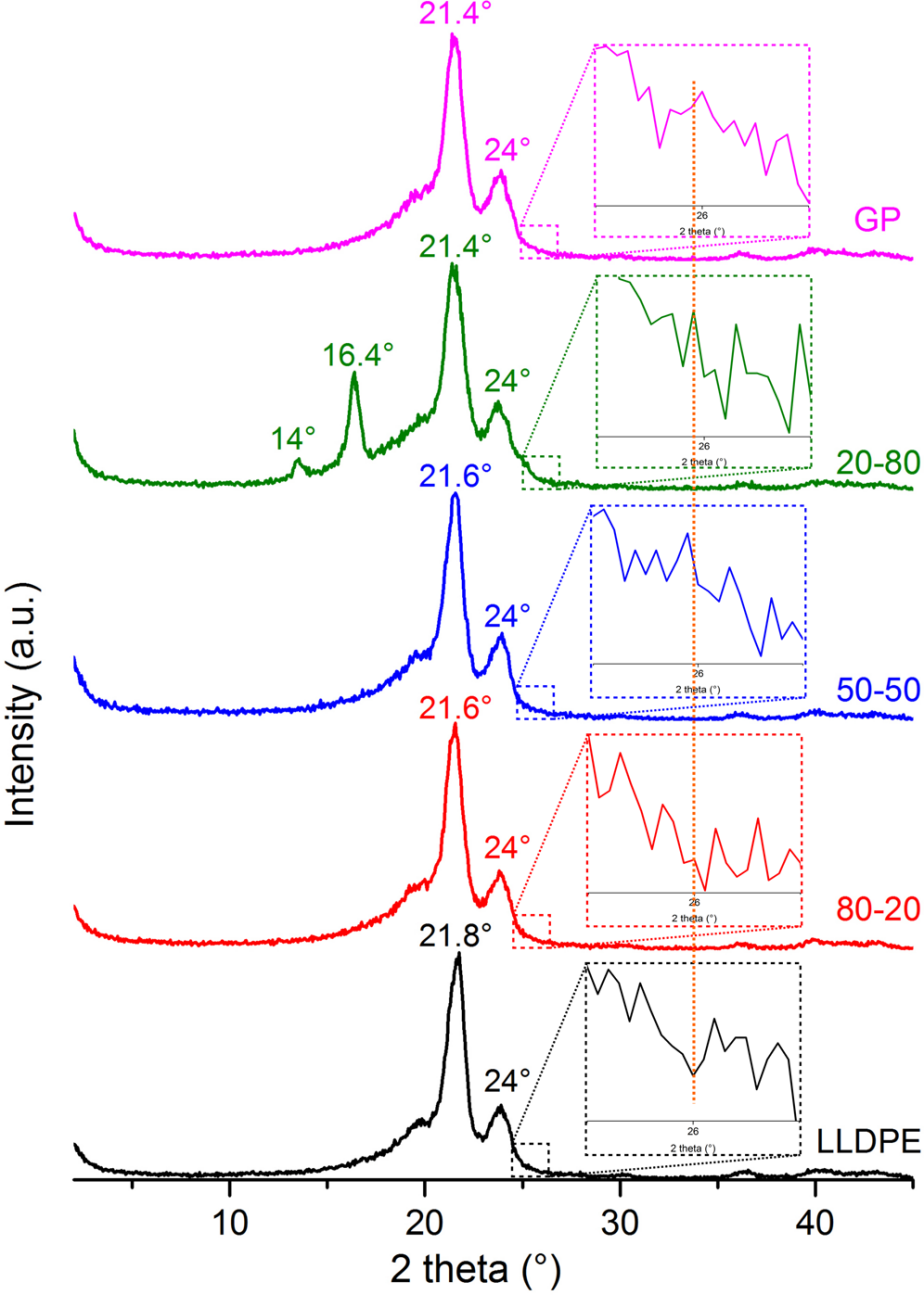
WAXD curves of the precursors and composites.

### Raman Spectroscopy

To better identify the vibration modes of GP matter, the Raman spectra were separated into two spectral regions: 3500–2500 and 1750–1000 cm^–1^ (Figure 11). All spectra presented the main vibrational modes of LLDPE named 2883 cm^–1^ (CH_2_, asymmetric stretching); 2850 cm^–1^ (CH_2_, symmetric stretching); 1460 and 1440 cm^–1^ (CH_2_, bending, amorphous phase); 1418 cm^–1^ (CH_2_, bending and CH_2_, wagging); 1305 and 1298 cm^–1^ (CH_2_, twisting); 1170 cm^–1^ (CH_2_, rocking); and 1130 and 1063 cm^–1^ (C–C, symmetric and asymmetric stretching).^41–43^ Since the amount of GP in the composites was very low, the spectra were deconvoluted in the regions of interest in order to identify the GP vibrations bands. It was possible to identify GP bands around 2700 cm^–1^ (GO), 1582 cm^–1^ (G), and around 1350 cm^–1^ named as disorder-induced D-band as published by Malard et al. (Figure 12).^44^

**Figure 11:**
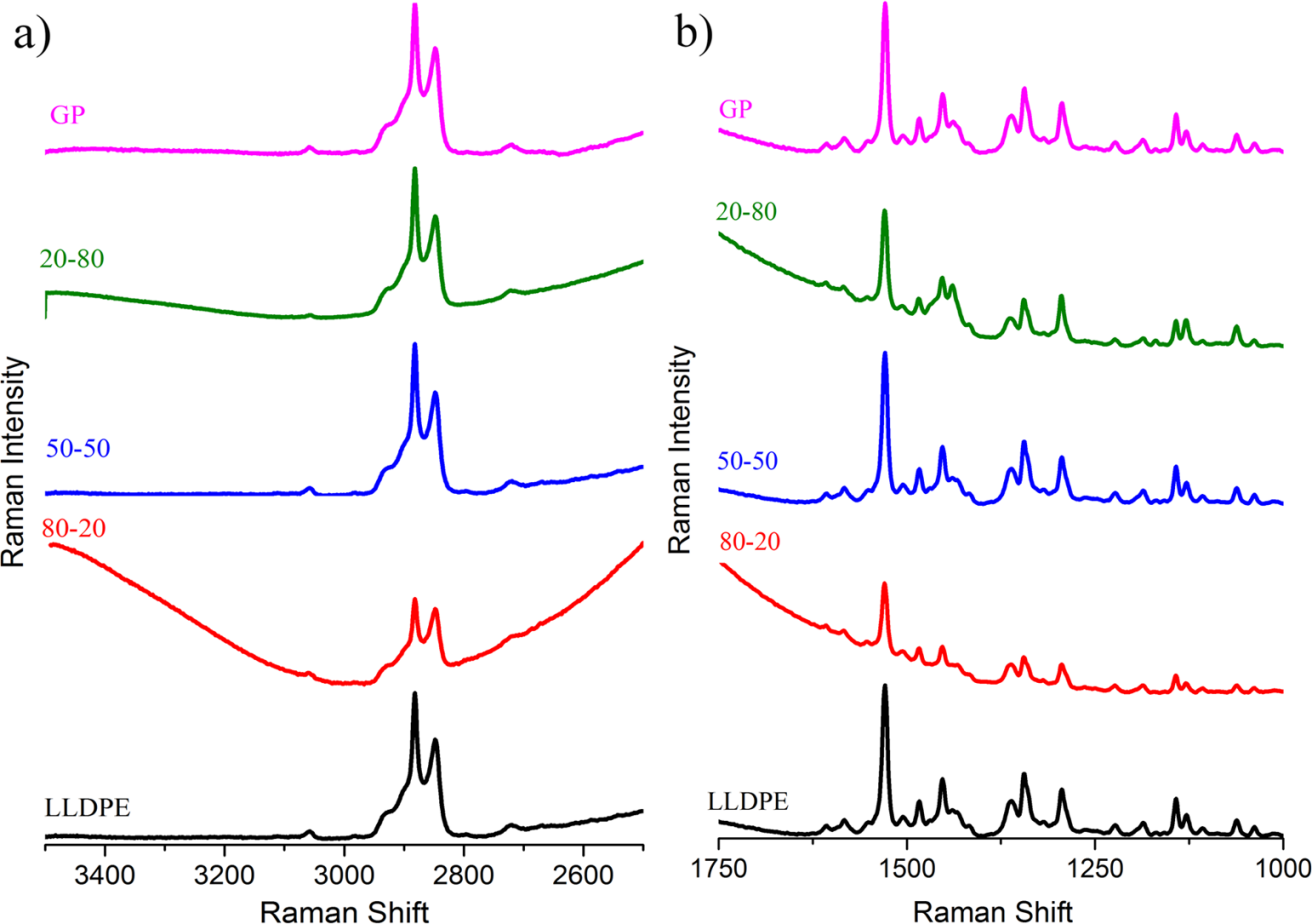
Raman spectra at two spectral regions: (a) 3500–2500 and (b) 1750–1000 cm^–1^.

**Figure 12:**
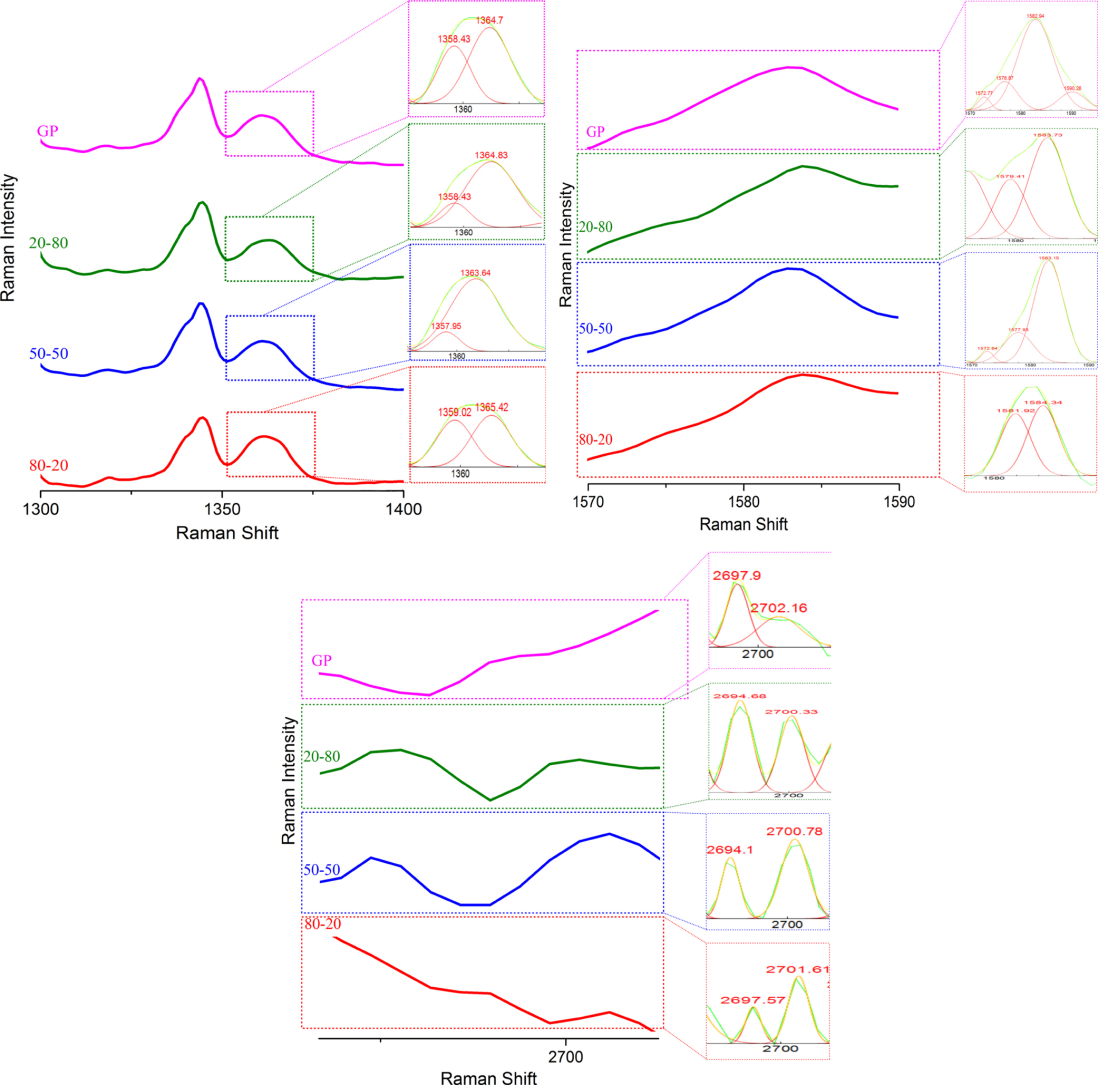
Deconvoluted spectral regions to identify GP bands.

### Melt Flow Rate

Figure 13 presents the MFR evaluation. Only from 20% of GP was noticed a progressive increase in MFR. Baumer et al. rotomolded specimens based on composite of polyethylene filled with calcium carbonate. The authors pointed out that no significant variation of MFR and degree of crystallinity were registered.^2^ Herein, the results seem to be within experimental error.

**Figure 13:**
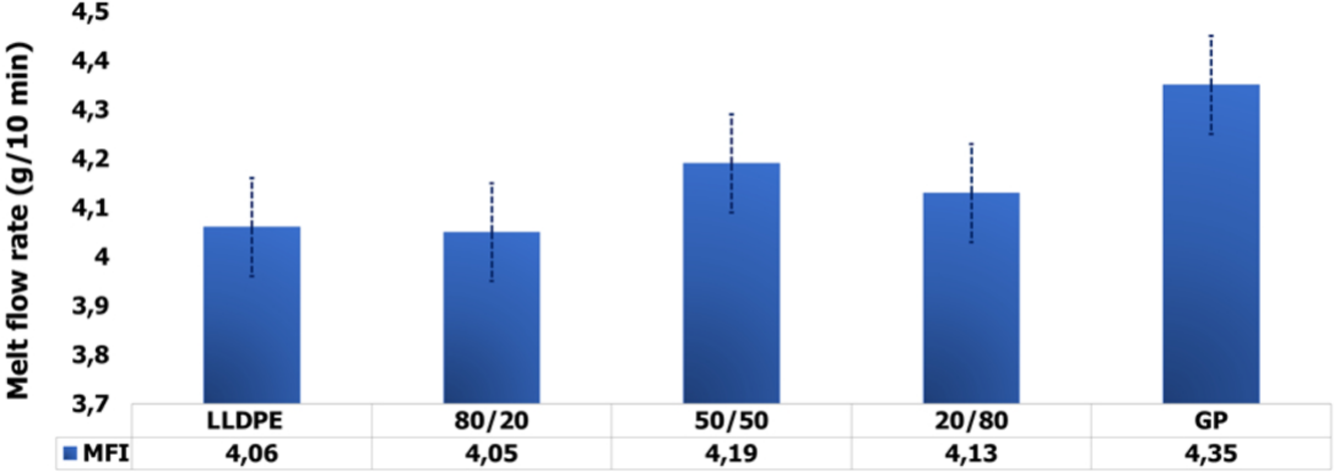
MFI data amplification of first and second heating curves of the precursors and composites.

### Rheology

Figure 14 shows the storage (*G*′) and loss (*G*″) moduli curves as a function of angular frequency. All curves are superimposed with profile suchlike to pseudoplastic material. Also, it is possible to infer that LLDPE presents physicochemical similarity to polymer-base of GP. Figure 15 displays the complex viscosity curves (η*) as a function of the angular frequency. All samples are overlapped, endorsing the pseudoplastic behavior and the resemblance of the material. Table 7 introduces the ratio *G*″/*G*′ of the samples at frequency of 100 Hz. The results revealed that at any composition, the addition of GP into LLDPE did not change its rheologic behavior endorsing the *G*′, *G*″ and viscosity evaluations.

**Figure 14:**
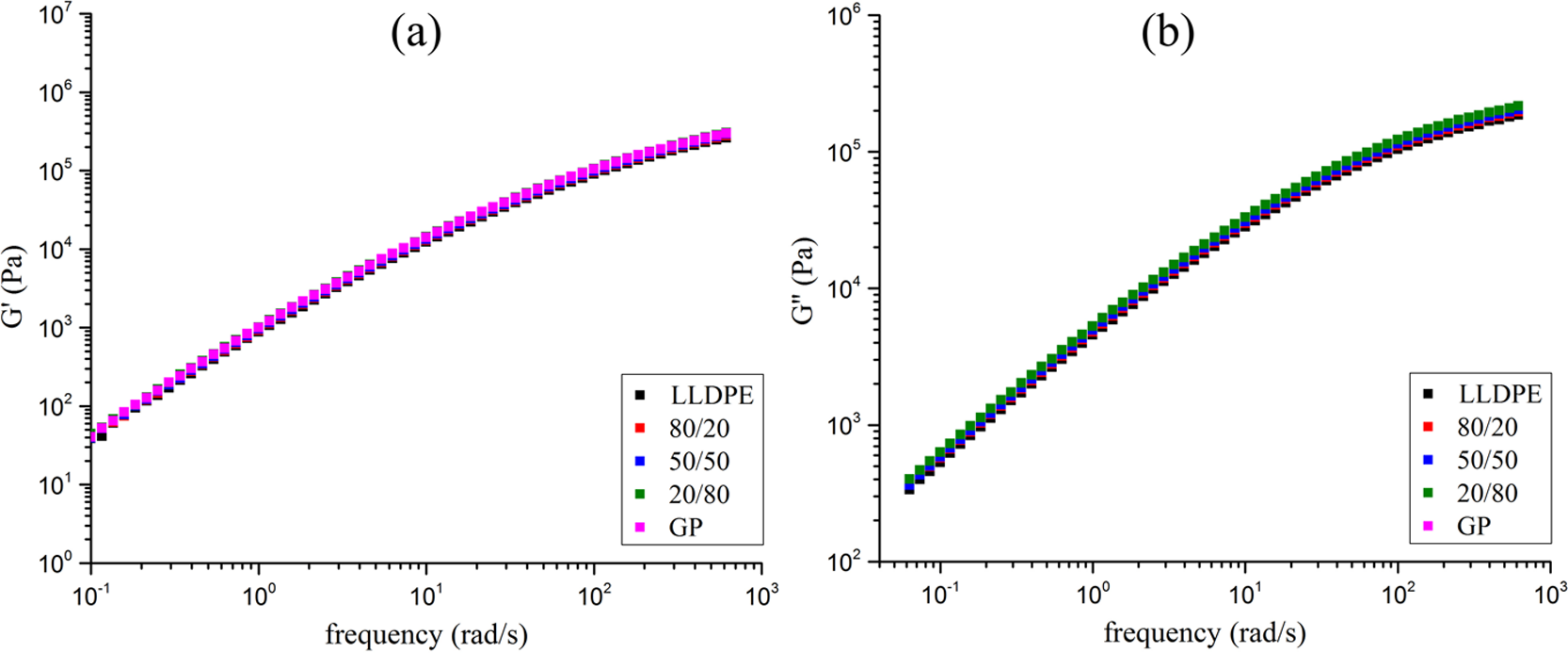
(a) *G*′ and (b) *G*″.

**Figure 15:**
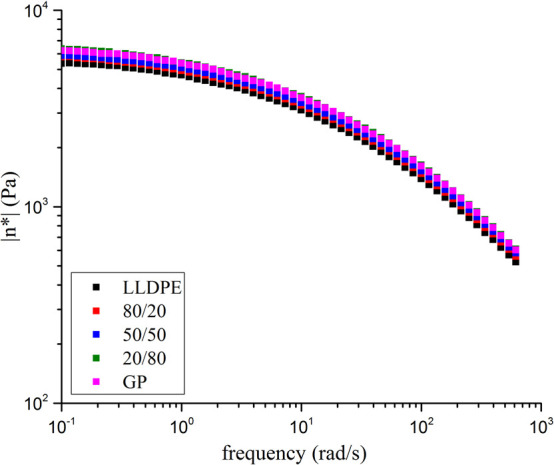
Complex viscosity of the samples.

**Table 7:** *G*′, *G*″ and *G*″/*G* Ratio of the Samples at a Frequency of 100 Hz

code	*G*′ (Pa)	*G*″ (Pa)	*G*″/*G*
LLDPE	90.790	104.500	1.151
80/20	96.320	110.900	1.151
50/50	99.190	114.500	1.154
20/80	106.400	122.800	1.154
GP	104.800	121.500	1.159

### Scanning Electron Microscopy

Figure 16 depicts SEM images of the precursors and composites. Also, the GP image (magnitude of 100,000) was inserted highlighting the GP matter (see red arrow). All images indicate ductile fracture related to thermoplastic material. Some striations were noticed in the LLDPE image due to the plastic deformation during the fracture process. This evidence was mostly viewed in the composites when the amount of GP increased.^45^ The effect shown by SEM images could be associated with the amorphization process of LLDPE chains with the increase in the GP content.

**Figure 16:**
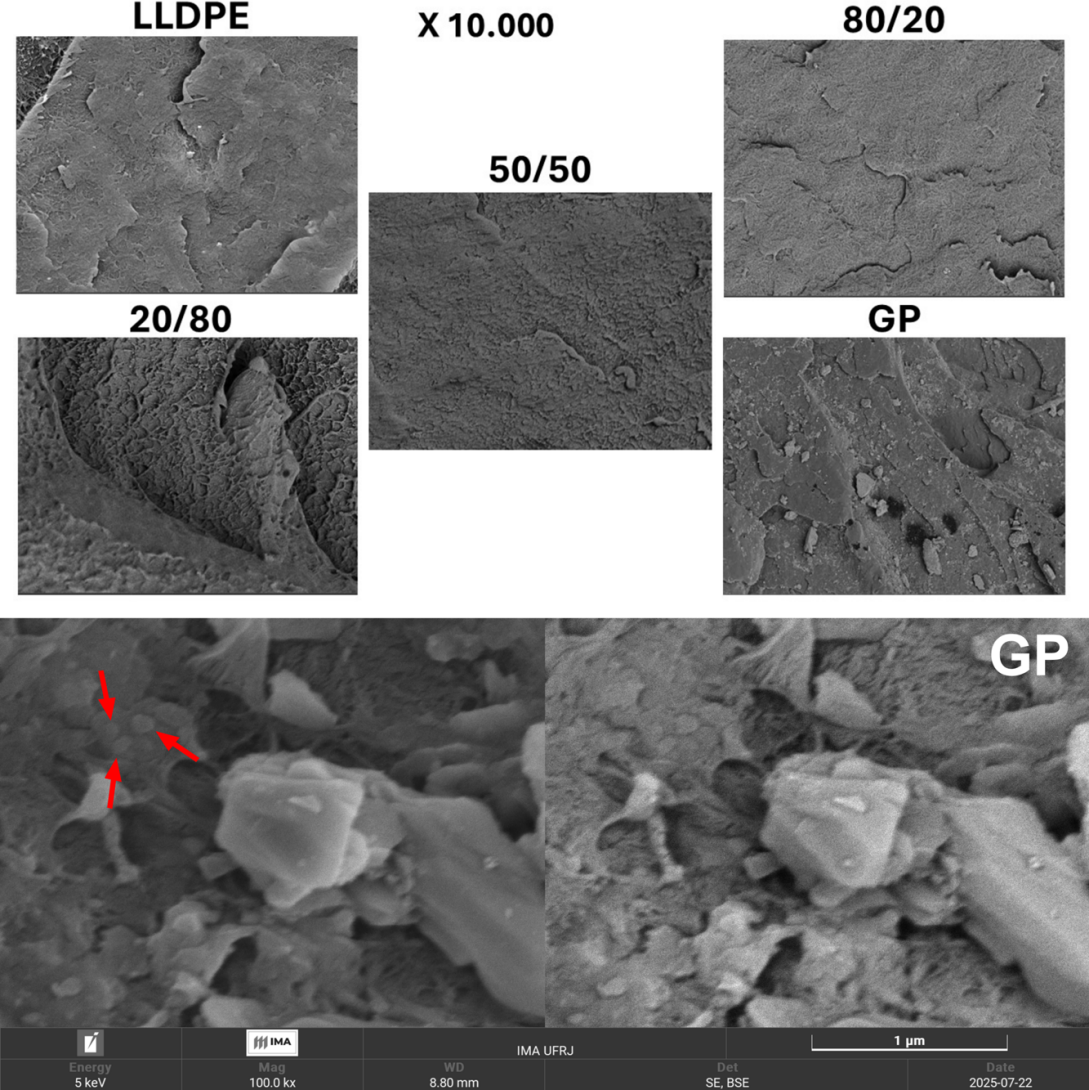
SEM images of LLDPE, GP, 80/20, 50/50, 20/80 (magnitude 10,000).

## Conclusion

This work developed LLDPE compounds with a very low GP content for rotational molding application. The rheological data were not affected by the presence of GP. A slight reduction in the processing time was revealed, mostly in the cooling cycle. There was an improvement in the impact strength of the rotationally molded sample. A decrease in the degree of crystallinity was noted when GP was added. The results were attributed to the effect of GP’s thermal conductivity. Even at low levels, GP helped thermally conduction along the polymer chains. The cooling cycle of the processing occurred more quickly, affecting the crystallization of LLDPE. Then, there was a decrease in the degree of crystallinity, which means that the amorphization of LLDPE led to the improvement of the impact behavior. In the current scientific literature, no works approaching LLDPE with a too low content of GP were found. Considering that the rotational molding is a process without pressure but with high energy cost, any approach concerning energy savings should be welcomed.

## Data Availability

The data sets generated during and/or analyzed during the current study are not publicly available (belong to my Institution—Universidade Federal Rio de Janeiro-UFRJ, Brazil).
